# Quantifying ventilation change due to radiation therapy using 4DCT Jacobian calculations

**DOI:** 10.1002/mp.13105

**Published:** 2018-08-31

**Authors:** Taylor J. Patton, Sarah E. Gerard, Wei Shao, Gary E. Christensen, Joseph M. Reinhardt, John E. Bayouth

**Affiliations:** ^1^ Department of Medical Physics University of Wisconsin – Madison Madison WI 53705 USA; ^2^ Department of Biomedical Engineering The University of Iowa Iowa City IA 52242 USA; ^3^ Department of Electrical and Computer Engineering The University of Iowa Iowa City IA 52242 USA; ^4^ Department of Human Oncology University of Wisconsin – Madison Madison WI 53792 USA

**Keywords:** 4DCT, deformable image registration, lung cancer, RT response, ventilation

## Abstract

**Purpose:**

Regional ventilation and its response to radiation dose can be estimated using four‐dimensional computed tomography (4DCT) and image registration. This study investigated the impact of radiation therapy (RT) on ventilation and the dependence of radiation‐induced ventilation change on pre‐RT ventilation derived from 4DCT.

**Methods and materials:**

Three 4DCT scans were acquired from each of 12 subjects: two scans before RT and one scan 3 months after RT. The 4DCT datasets were used to generate the pre‐RT and post‐RT ventilation maps by registering the inhale phase image to the exhale phase image and computing the Jacobian determinant of the resulting transformation. The ventilation change between pre‐RT and post‐RT was calculated by taking a ratio of the post‐RT Jacobian map to the pre‐RT Jacobian map. The voxel‐wise ventilation change between pre‐ and post‐RT was investigated as a function of dose and pre‐RT ventilation.

**Results:**

Lung regions receiving over 20 Gy exhibited a significant decrease in function (3.3%, *P* < 0.01) compared to those receiving less than 20 Gy. When the voxels were stratified into high and low pre‐RT function by thresholding the Jacobian map at 10% volume expansion (Jacobian = 1.1), high‐function voxels exhibited 4.8% reduction in function for voxels receiving over 20 Gy, a significantly greater decline (*P* = 0.037) than the 2.4% reduction in function for low‐function voxels. Ventilation decreased linearly with dose in both high‐function and low‐function regions. High‐function regions showed a significantly larger decline in ventilation (*P* ≪ 0.001) as dose increased (1.4% ventilation reduction/10 Gy) compared to low‐function regions (0.3% ventilation reduction/10 Gy). With further stratification of pre‐RT ventilation, voxels exhibited increasing dose‐dependent ventilation reduction with increasing pre‐RT ventilation, with the largest pre‐RT Jacobian bin (pre‐RT Jacobian between 1.5 and 1.6) exhibiting a ventilation reduction of 4.8% per 10 Gy.

**Conclusions:**

Significant ventilation reductions were measured after radiation therapy treatments, and were dependent on the dose delivered to the tissue and the pre‐RT ventilation of the tissue. For a fixed radiation dose, lung tissue with high pre‐RT ventilation experienced larger decreases in post‐RT ventilation than lung tissue with low pre‐RT ventilation.

## 1 Introduction

Lung cancer is the most common cancer worldwide, and the third most common cancer in the United States.^1^, ^2^ Patients treated with radiotherapy (RT) have a 5% to 20% occurrence rate of RT‐induced lung injury.^3^ Radiation pneumonitis has been shown to increase in prevalence with increased cumulative lung dose, increased age, and an inferior‐located tumor.^4^, ^5^ Furthermore, it has been shown that radiation‐induced lung injury may be influenced by patients’ pre‐RT lung function.^6^, ^7^

### 1.A. 4DCT‐derived ventilation

Ventilation, along with blood perfusion and gas exchange, are necessary components of lung function. Four‐dimensional computed tomography (4DCT) can be used to compute a surrogate for regional ventilation of lung tissue and provide a spatial map of the local lung tissue expansion and contraction. The term ventilation in this paper refers to this tissue expansion surrogate. The advantage of using 4DCT data to calculate ventilation is that a 4DCT scan is typically acquired as part of the treatment planning process. In addition, 4DCT‐derived ventilation maps have a high‐spatial resolution and show the temporal changes of the lung over different phases of the breathing cycle. Reinhardt et al.^8^ calculated ventilation from CT image volumes using the Jacobian determinant of the transformation calculated by deformable image registration. Simon^9^ and Guerrero et al.^10^ used density changes caused by the increase in air in the lungs to calculate ventilation. Kipritidis et al.^11^ omits image registration and uses the intensity values of the phase‐averaged 4DCT dataset to calculate ventilation. These ventilation calculations have been compared to clinically accepted measures of pulmonary function: Xenon CT, pulmonary function tests, and Galligas PET imaging, respectively.^11^, ^12^, ^13^ This paper estimates ventilation using the Jacobian determinant of the transformation.

### 1.B. Dose dependence of lung function

Many studies have calculated functional change in the lungs during or after radiation therapy using different modalities. Some studies^14^, ^15^, ^16^ assessed changes found in pulmonary function tests (PFT), which represent changes in the global function of the lung. Yuan et al. studied changes during RT and found no significant changes in PFT metrics. Vagane et al. and Guerra et al. showed no change in forced vital capacity or forced expiratory volume during the first second divided by vital capacity, but Guerra saw that the diffusing capacity of the lungs for carbon monoxide was reduced in a majority of patients. Non‐significant results in global PFT measures suggest looking at regional lung function metrics, which can be based on single‐photon emission computed tomography (SPECT) or 4DCT data. Yuan et al. showed that during treatment, ventilation/perfusion improved significantly.^16^ Farr et al. and Boersma et al.^17^, ^18^ looked at the change in SPECT data from pre‐RT to 5 and 3 months post‐RT, respectively. Both found that lung function decreased as dose to the lung region increased. Seppenwoolde et al.^19^ compared the radiation‐induced SPECT changes in non‐small cell lung cancer (NSCLC) patients to a reference group of non‐lung cancers. They found that in well‐perfused lung regions, the NSCLC patients responded similar to the reference group, but in poorly perfused regions, there was less decrease in function due to local reperfusion. Vinogradskiy et al.^20^ studied 4DCT‐based functional change during radiation therapy and found no apparent relationship between ventilation and dose. Ding et al.^21^ used 4DCT data to study the function change pre‐RT to post‐RT and found that there was a decrease in function for regions receiving greater than 24 Gy, and had varied responses in regions with less than 24 Gy. King et al.^22^ analyzed pre‐RT and post‐RT 4DCT datasets and found a significant dose‐dependent decline in function for voxels receiving over 20 Gy. To our knowledge, this is the first study to examine if the radiation dose response is dependent on 4DCT‐derived regional lung function prior to therapy. The purpose of this study was to quantitate the change in regional ventilation after a course of radiation therapy, and to establish its dependence on pre‐RT ventilation as derived from 4DCT datasets along with deformable image registration.

## 2 Materials and methods

### 2.A. 4DCT acquisition

This institutional review board‐approved prospective study (NCT01039649) evaluated twelve lung cancer subjects who underwent radiation therapy treatment. Exclusion criteria included prior or future planned surgery for treatment of the lung cancer, prior thoracic radiotherapy, uncontrolled intercurrent illness, pregnancy, severe COPD, underlying collagen vascular disease, oxygen dependence, recent abdominal surgery, and being under 18 yr old. The patient information for the study cohort is shown in Table 1. Two patients had early stage disease (I, II) and ten had later stage disease (III, IV). There was one patient treated with hypofractionation and the rest were treated with 1.8–2 Gy fractions. Prior to treatment, each subject had a 3D breath‐hold CT image acquired, which was used for radiation treatment planning. Three 4DCT scans were acquired for each subject: two scans before RT, with a 5–15 min break between scans, and one scan 3 months after completion of RT. The 4DCT datasets were reconstructed into 10 phases. All scans were acquired in helical mode with a 2 mm slice thickness, 0.5 mm slice increment, 0.5 s tube rotation time, 0.1 pitch, 120 kVp, 700 mAs, and 1.2 mm beam collimation on a Siemens Biograph 30‐slice CT scanner with subjects in the supine position. The scanner used an Anzai AZ‐773V with a strain gauge belt as a surrogate for the respiratory pattern. To increase the repeatability of the respiratory pattern, the subjects were guided with audible timing cues, which were played throughout the scan (RESP@RATE, Intercure Ltd., Lod Israel). The subjects in this study were selected from a protocol with a larger cohort; those excluded either withdrew prior to completion of the follow‐up scans or were missing datasets (31), or provided 4DCT scans with major artifacts (7).

**Table 1:** Patient information for study cohort. Disease acronyms are malignant peripheral nerve sheath tumor (MPNST), non‐small cell lung cancer (NSCLC), and small cell lung cancer (SCLC)

Subject	Sex	Age	Disease	Stage	Prescription dose [Gy]	Number of Fx	Karnofsky performance status
1	F	30	MPNST	IV	50	5	90
2	M	72	NSCLC	IIIB	70	35	80
3	M	47	Lymphoma	III	36	20	90
4	F	47	Lymphoma	I	36	20	80
5	M	78	SCLC	IV	61.2	34	90
6	F	38	SCLC	IV	61.2	34	90
7	F	69	NSCLC	IIIA	70	35	80
8	M	69	NSCLC	I	70	35	90
9	F	63	NSCLC	IV	60	30	80
10	M	58	NSCLC	IIIB	65	35	100
11	F	68	NSCLC	IIIA	64	32	90
12	M	55	NSCLC	IV	66	33	80

### 2.B. Data preprocessing

Deformable image registration was used to find the pointwise correspondence between inspiration and expiration phase images. The registration algorithm uses a multi‐resolution cubic B‐spline parameterization and a sum of squared tissue volume difference (SSTVD) similarity metric. The Jacobian determinant of this transformation was used to estimate local ventilation. The registration accuracy was validated using anatomical landmarks,^23^ and the Jacobian ventilation calculation was validated by comparison to Xenon CT scans on ventilated sheep.^8^ A Jacobian value equal to one represents no local volume change, greater than one represents local expansion, and less than one represents local contraction of the lung tissue. This study used equivalent tidal volumes (ETV) for phase selection, which has been shown to increase the repeatability of the ventilation measurement, to isolate the effect of radiation in longitudinal changes.^24^ A 4DCT phase image at the beginning of inhalation was selected as the fixed image, and an image at the end of inhalation was selected as the moving image. The specific fixed and moving images were chosen to meet a 100 cc tidal volume range between all three scans with the goal of normalizing a subject's effort longitudinally.

### 2.C. Overview of Jacobian analysis

**Figure 1:**
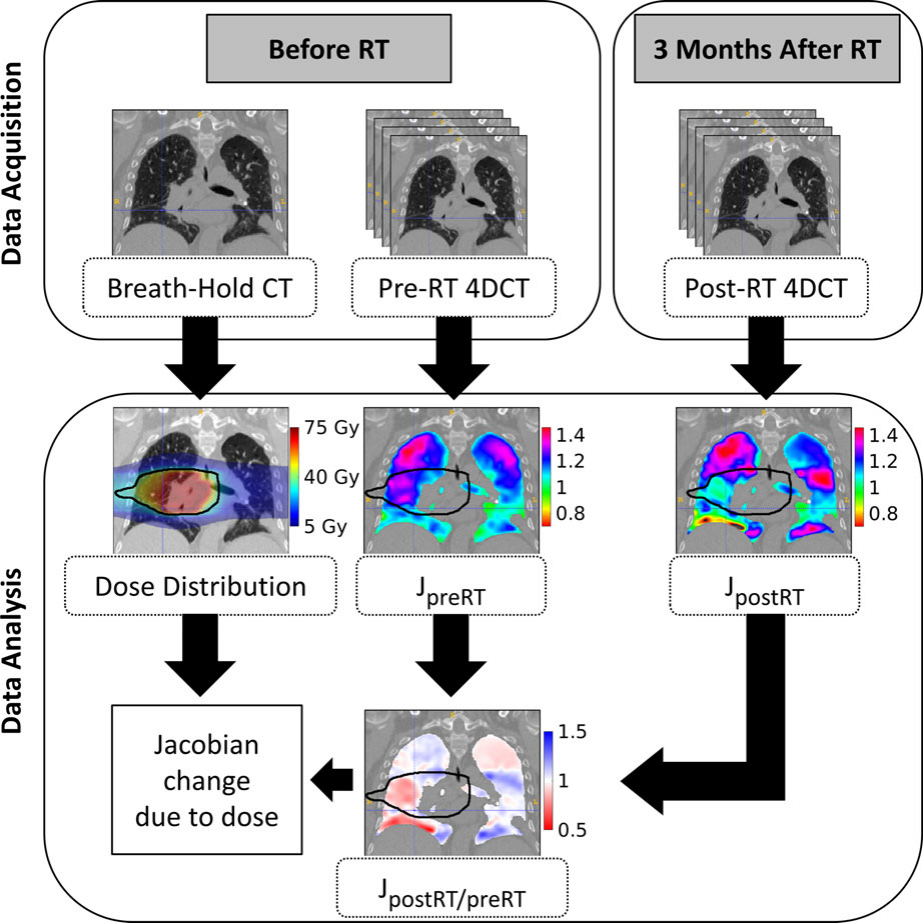
Diagram of the process used to measure the Jacobian change due to dose. A breath‐hold CT scan was acquired before RT, which was used to plan the dose distribution. Repeat 4DCT datasets were acquired before RT and one dataset was acquired after RT; these were used to calculate the pre‐RT and post‐RT Jacobian, respectively. A Jacobian ratio map was obtained by taking the voxel‐wise ratio of post‐RT Jacobian to pre‐RT Jacobian. The Jacobian ratio map was analyzed with the dose distribution to calculate the dose dependence of ventilation.^24^ The 30 Gy isodose line is outlined in black.^27^

An overview of the Jacobian calculations and subsequent analysis of ventilation change for one subject can be seen in Fig. 1. Before RT, an expiratory breath‐hold 3DCT and two 4DCTs were acquired. The dose distribution was planned on the 3D breath‐hold CT since it was a higher quality image and free of motion artifacts. For subjects with both pre‐RT 4DCT images fitting the ETV criteria, the two pre‐RT Jacobian maps were deformably registered to the 3D breath‐hold CT and then geometrically averaged to get a more robust estimate of each subject's pre‐RT ventilation, J_preRT_. For subjects with only one pre‐RT 4DCT image satisfying the volume constraints, this single Jacobian map was used as the estimate of their pre‐RT ventilation, J_preRT_. Three months after completion of RT, another 4DCT was acquired and the Jacobian map was calculated to estimate the post‐RT ventilation, J_postRT_. To visualize the consequences of the delivered dose, J_preRT_ and J_postRT_ were each deformed to the common coordinate space of the 3D breath‐hold CT. A Jacobian ratio map was calculated by taking a voxel‐wise ratio of J_postRT_ to J_preRT_. This ratio map, J_postRT/preRT_, quantitates the fractional change in ventilation due to RT. A ratio greater than one represents an increase in local ventilation post‐RT, and a ratio less than one represents a reduction in local ventilation. J_postRT/preRT_ was used in conjunction with the dose information from treatment to assess dose response at the voxel level. The 4DCT datasets, originally 2 × 0.98 × 0.98 mm, and dose distributions, originally 4 × 4 × 4 mm, were resampled to 1 × 1 × 1 mm for voxel‐wise comparison.

### 2.D. Determining response to radiation therapy and pre‐RT ventilation dependence

Dose response was assessed by first determining the calculated radiation dose delivered to each voxel of tissue within the lung. Doses were calculated for a heterogeneous medium derived from the CT dataset using a convolution superposition algorithm (Philips Pinnacle,^3^ Fitchburg, WI, USA), adjusted to perform adaptive sampling during convolution dose calculation. Specifically, the dose algorithm sampling was relaxed to every fourth point in the 4 mm × 4 mm dose grid in areas of flat dose. As described above, J_postRT/preRT_ provided the fractional change in ventilation following RT. This response map was deformed into the coordinate system of the computed dose and analyzed to establish the dose‐response relationship. Since there are several fractionation schemes, all delivered doses have been converted to equivalent dose in 2 Gy fractions (EQD2). The *α*/*β* of radiation pneumonitis is 4 and this value is used for the effects in this study due to the time to onset of pneumonitis corresponding with the 3‐month post‐RT time point.^25^

J_postRT/preRT_ and EQD2 were analyzed to quantitate the ventilation change due to dose. The magnitude of the dose response was assessed for all voxels within the lung, excluding those in the gross tumor volume. All voxels were stratified by a dose threshold of 20 Gy, which was defined due to its importance in treatment planning. The average J_postRT/preRT_ was calculated for the voxels in each of the two dose bins for each subject. The mean J_postRT/preRT_ per dose bin was then averaged over all subjects to quantitate the decrease in function between low‐dose and high‐dose tissues. The voxels in each dose bin were further stratified into two functional bins defined by a pre‐RT Jacobian threshold, creating four groups of voxels. This functional stratification was performed to determine if the dose response was also dependent on pre‐RT Jacobian. To establish a functional threshold, the complementary cumulative distribution of J_preRT_ was created for each subject. The median pre‐RT Jacobian for all subjects was 1.1, and was chosen as the threshold between high function and low function. The average Jacobian ratio was calculated for each of the four bins for each subject. The decline in function due to dose was calculated by the difference between the average of the high‐dose and low‐dose voxels for each functional stratification. To establish the significance of pre‐RT function, the Student's *t* test was used to compare the functional decline of the low‐function voxels to that of the high‐function voxels.

Keeping constant the two functional bins, the dose was further stratified from two dose bins into twelve dose bins of 5 Gy from 0 to 60 Gy to analyze the linear trend of J_postRT/preRT_ for both high‐ and low‐function lung tissue. Linear regression was performed on the two groups of data. The homogeneity of regression was tested using analysis of covariance to determine if there was a significant difference in the dose response between high‐ and low‐function voxels. Regression slopes were also tested for significance using analysis of variance. Cumulative dose‐volume histograms of the combined ipsilateral and contralateral lungs excluding the gross tumor volume were created to visualize the dose distributions and the relative volumetric extent of potential damage at different dose levels.

Finally, keeping constant the twelve dose bins, the voxels were further divided from two functional bins into six pre‐RT function bins of equal size from 1 to 1.6. This further stratification allowed the analysis of functional decline for more discrete groups of pre‐RT function. Linear regression was performed for each pre‐RT Jacobian bin looking at the response of J_postRT/preRT_ to increasing dose. The slopes of these lines quantitate the lung tissue's sensitivity to dose and were tabulated for each pre‐RT Jacobian.

## 3 Results

### 3.A. Radiation‐induced changes

**Figure 2:**
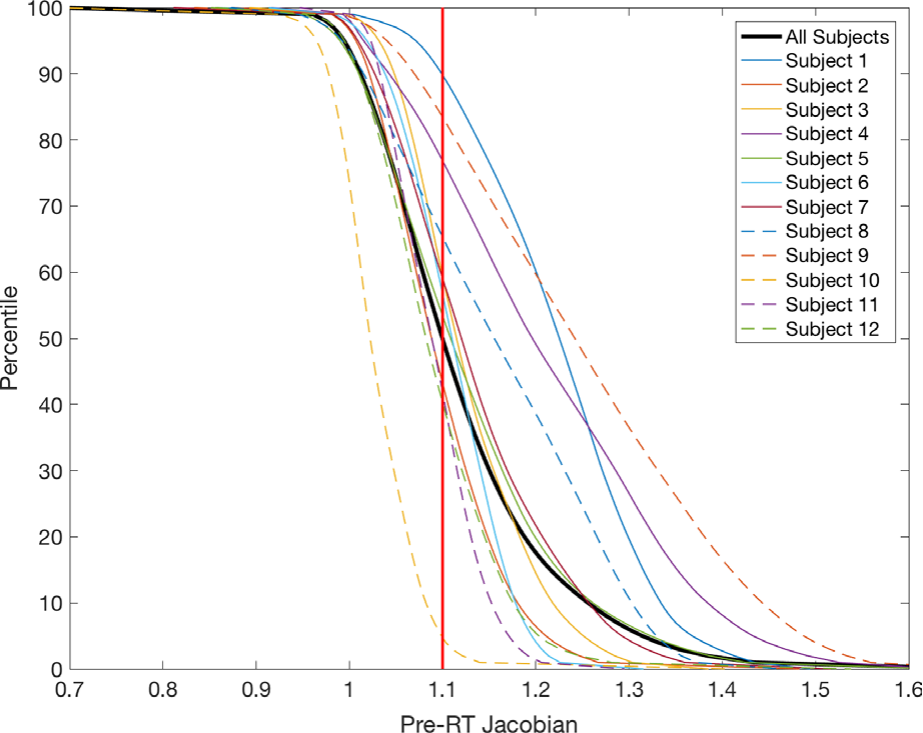
Complementary cumulative distribution, P(X > x), of J_pre_ _RT_ for all subjects.

The J_postRT/preRT_ average for all subjects for voxels receiving less than 20 Gy was 1.014 ± 0.018 and 0.981 ± 0.024 for voxels receiving more than 20 Gy, resulting in a significant (*P* < 0.01) J_postRT/preRT_ decline of 0.033 for high‐dose voxels. To determine the amount of lung tissue that expands by more than 10%, the complementary cumulative distributions of J_preRT_ for all subjects were examined (see Fig. 2).

**Figure 3:**
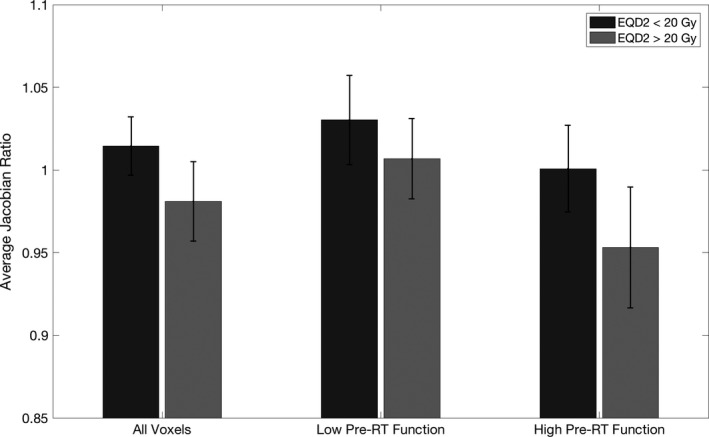
Bar plot of the average J_post_ _RT__/pre_ _RT_ across subjects for voxels receiving <20 Gy and >20 Gy for all voxels, voxels with low pre‐RT function, and voxels with high pre‐RT function. Error bars represent one standard deviation above and below the mean across subjects.

The median for all subjects, represented by the vertical red line, was 1.1, and is used to represent the threshold between high function and low function. The response between the high‐dose and low‐dose tissue for all voxels, low‐function voxels, and high‐function voxels is pictured in Fig. 3.

The average J_postRT/preRT_ for high and low pre‐RT function and high‐ and low‐dose groups for each subject are shown in Table 2. On average low‐function voxels receiving low dose had a J_postRT/preRT_ of 1.030 ± 0.027 while low‐function voxels receiving high dose had an average J_postRT/preRT_ of 1.007 ± 0.024, resulting in a relative decrease in J_postRT/preRT_ of 0.024 for voxels receiving more than 20 Gy. Similarly, high‐function voxels receiving low dose versus high dose had an average J_postRT/preRT_ of 1.001 ± 0.026 and 0.953 ± 0.036, respectively, resulting in a relative decrease of 0.048. The difference between J_postRT/preRT_ of high‐ and low‐dose voxels was significantly higher for high‐function voxels (*P* = 0.037), signifying an increased sensitivity to dose for tissue with high pre‐RT function.

**Table 2:** Average J_postRT/preRT_ for each dose and pre‐RT Jacobian bin for each subject with standard deviation in parentheses. Difference between the <20 Gy dose bin and >20 Gy dose bin for each functional stratification on right. The difference between <20 Gy and >20 Gy for high pre‐RT function is significantly different from that of low pre‐RT function, *P* = 0.037

	Average Jacobian ratios				Difference between <20 Gy and >20 Gy	
	Low pre‐RT function		High pre‐RT function			
	<20 Gy	>20 Gy	<20 Gy	>20 Gy	Low Pre‐RT	High Pre‐RT
Subject 1	1.004 (0.053)	0.988 (0.088)	0.993 (0.062)	0.942 (0.075)	0.017 (0.062)	0.052 (0.078)
Subject 2	1.016 (0.052)	0.991 (0.036)	1.013 (0.067)	0.956 (0.045)	0.025 (0.059)	0.058 (0.044)
Subject 3	1.022 (0.064)	0.970 (0.032)	0.984 (0.069)	0.926 (0.046)	0.052 (0.070)	0.058 (0.044)
Subject 4	1.026 (0.055)	0.990 (0.037)	1.016 (0.075)	0.993 (0.082)	0.036 (0.073)	0.023 (0.062)
Subject 5	1.091 (0.114)	1.048 (0.095)	1.007 (0.128)	0.963 (0.081)	0.043 (0.129)	0.044 (0.099)
Subject 6	1.029 (0.054)	1.003 (0.035)	0.987 (0.048)	0.959 (0.041)	0.026 (0.055)	0.028 (0.044)
Subject 7	1.022 (0.041)	0.996 (0.045)	1.010 (0.056)	0.944 (0.057)	0.026 (0.052)	0.066 (0.056)
Subject 8	1.018 (0.122)	1.041 (0.078)	0.984 (0.075)	0.940 (0.096)	−0.022 (0.095)	0.044 (0.102)
Subject 9	1.079 (0.126)	1.040 (0.067)	1.048 (0.100)	0.912 (0.123)	0.039 (0.104)	0.136 (0.124)
Subject 10	1.005 (0.053)	0.997 (0.044)	0.941 (0.044)	0.889 (0.041)	0.008 (0.054)	0.052 (0.046)
Subject 11	1.018 (0.046)	1.004 (0.026)	1.021 (0.064)	1.004 (0.033)	0.014 (0.056)	0.017 (0.027)
Subject 12	1.034 (0.064)	1.014 (0.049)	1.005 (0.069)	1.011 (0.081)	0.020 (0.068)	−0.006 (0.056)
Average	1.030 (0.027)	1.007 (0.024)	1.001 (0.026)	0.953 (0.036)	0.024 (0.019)	0.048 (0.035)

### 3.B. Dose dependence of ventilation

**Figure 4:**
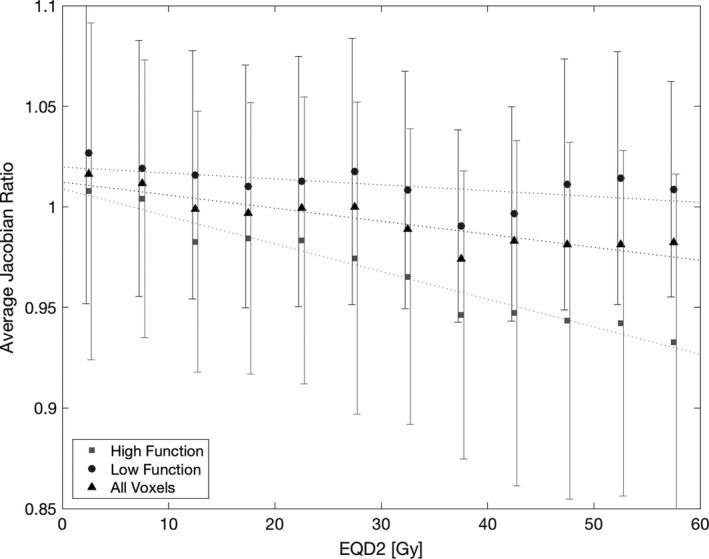
Average J_post_ _RT__/pre_ _RT_ from 0 to 60 Gy for all subjects, stratified by pre‐RT function. The pre‐RT Jacobian threshold between high function and low function was 1.1. Error bars represent values within one standard deviation of the mean.

Figure 4 shows the change in J_postRT/preRT_ as dose increases, categorized into regions of lung with high function and low function. Both the high function and low‐function trends are fairly linear, indicating an increasing reduction in lung tissue elasticity with increasing radiation dose. The low‐function voxels start at 0 Gy with a modest increase in function of 1.02 and end at 60 Gy with a ratio of one. The low‐function voxels have a very slight downward trend as dose increases, with a 0.3% ± 0.2% decrease in J_postRT/preRT_ per 10 Gy delivered dose. The high‐function lung voxels also started above a ratio of 1 at 0 Gy with an average Jacobian ratio of 1.01 decreasing to 0.93 at 60 Gy. The high‐function voxels showed a larger decrease in function over the range of doses, with a 1.4% ± 0.1% decrease in J_postRT/preRT_ per 10 Gy delivered dose. The regressions between the Jacobian ratios of high‐function and low‐function voxels are significantly different (*P* ≪ 0.001) calculated with analysis of covariance. Both slopes were also significantly different from zero (*P* ≪ 0.001) calculated using analysis of variance.

**Figure 5:**
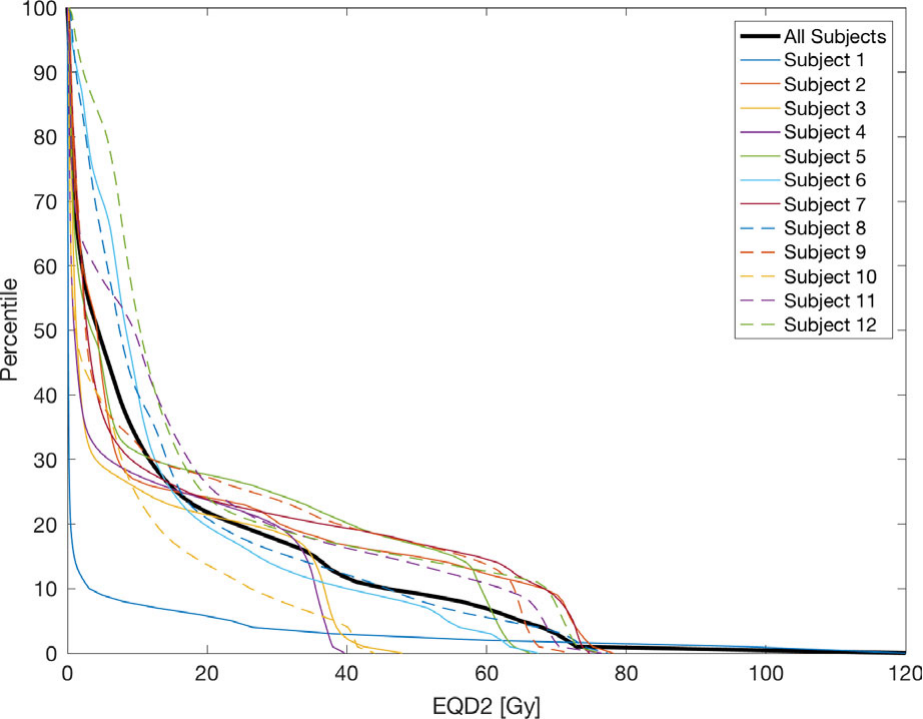
Cumulative dose‐volume histograms of combined ipsilateral and contralateral lung excluding the GTV for all subjects.

**Figure 6:**
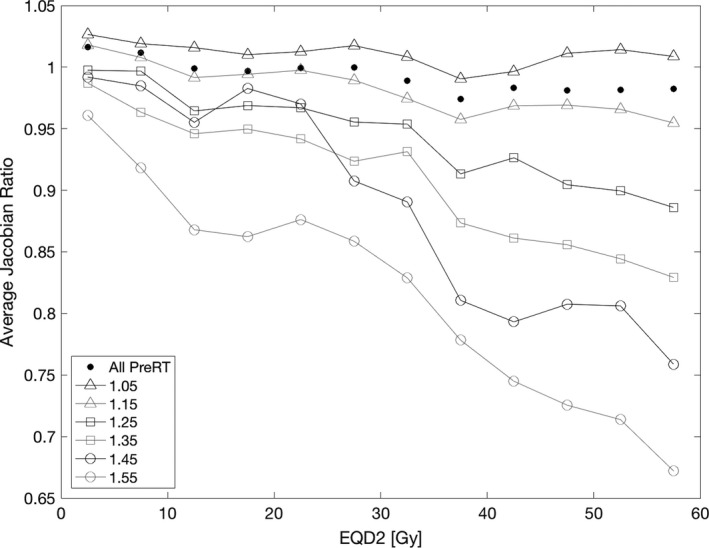
Average J_post_ _RT__/pre_ _RT_ from 0 to 60 Gy for all subjects, grouped by pre‐RT function. The pre‐RT Jacobian was stratified into six equally sized bins between 1 and 1.6; the nominal bin centers represent the specific stratification of the line.

Figure 5 shows the cumulative dose‐volume histograms of both ipsilateral and contralateral lung for the 12 subjects. Although there are a minority of voxels in the high‐dose region, where the separation between high‐ and low‐function voxels is most apparent, there are 12% of voxels over 40 Gy, an average of 443 cc per subject, which corresponds to a 5% reduction in function of high‐function voxels compared to low‐function voxels. Figure 6 shows the functional response with continued stratification of pre‐RT function into six discrete bins of width 0.1. The volume of lung in each of these bins used for averaging is shown in Table 3.

**Table 3:** Volume of lung used to calculate average Jacobian ratio for each combination of pre‐RT Jacobian and dose plotted in Fig. 6

Pre‐RT Bin Center	Volume of lung in each bin [cc]											
	Dose bin center [Gy]											
	2.5	7.5	12.5	17.5	22.5	27.5	32.5	37.5	42.5	47.5	52.5	57.5
1.05	10593	3371	1680	832	552	551	566	1006	535	222	238	2439
1.15	7429	2449	1249	621	292	270	337	484	146	126	149	1005
1.25	3385	651	397	174	84	64	89	76	34	34	35	218
1.35	1473	103	88	89	56	39	29	18	13	10	10	43
1.45	476	18	14	9	10	7	8	8	5	4	3	21
1.55	104	4	2	2	3	3	4	3	3	2	2	14

At high doses, the separation between the response of the pre‐RT Jacobian bins shows that higher functioning tissue incurs more radiation‐induced damage. Linear regression was performed for each pre‐RT Jacobian bin as a function of J_postRT/preRT_ and dose. The changes in J_postRT/preRT_ with respect to dose are derived from the linear regression slopes and are presented in Table 4. The decline in J_postRT/preRT_ increases monotonically with pre‐RT Jacobian from a decline of 0.3% ± 0.2% per 10 Gy for the lowest functioning tissue to a decline of 4.8% ± 0.4% per 10 Gy for the highest functioning tissue.

**Table 4:** The relative J_postRT/preRT_ decrease per 10 Gy for each pre‐RT Jacobian stratification. The percent decrease in J_postRT/preRT_ was calculated using the lines of best fit through the data shown in Fig. 6

Pre‐RT Jacobian bin center	1.05	1.15	1.25	1.35	1.45	1.55
Jacobian ratio decrease per 10 Gy	0.3% ± 0.2%	1.0% ± 0.1%	2.0% ± 0.2%	2.9% ± 0.2%	4.7% ± 0.5%	4.8% ± 0.4%

## 4 Discussion

For this study, high‐function regions of the lung had a significantly greater radiation‐induced decline in function post‐RT than low‐function regions. We found that ventilation linearly decreases with dose, and there was no specific dose threshold between 0 and 60 Gy where damage peaks. This dose‐response linearity supplements the use of specific dose thresholds during treatment planning, for example, 20 Gy. Dose reduction to lung tissue was beneficial to all dose levels evaluated, and was increasingly important for regions of the lung with high ventilation. To our knowledge, this is the first evidence showing that 4DCT‐derived ventilation changes after radiotherapy treatments are dependent on both the delivered dose and the pre‐RT lung function at the voxel level. Several studies have shown that lung function is affected by dose using SPECT, 4DCT image registration, and CT densities.^14^, ^15^, ^16^, ^17^, ^18^, ^19^, ^20^, ^21^, ^22^ These studies generally agree that there is decreasing regional lung function with increasing dose delivered to the lung. King et al.^22^ reported a decline in average Jacobian of 1.8% for voxels between 20 and 40 Gy and 4.6% for voxels over 40 Gy. Our study found a decline in average Jacobian of 3.3% for all voxels over 20 Gy, which corroborates their findings, adding that this decline is also dependent on pre‐RT lung function with low‐function regions declining 2.4% and high‐function regions declining 4.8%. The dependence on pre‐RT function is further explored with a finer stratification of doses. The slope of the dose‐response curve is greater for regions of high pre‐RT ventilation compared to those with low ventilation. This finding agrees with similar studies of SPECT and PET lung function measures and their response to dose: high‐function regions have a greater response to dose than low‐function regions.^19^, ^26^

Prior work has shown the repeatability of ventilation estimates is improved by implementing the transformation‐based Jacobian calculation instead of the intensity‐based calculation, and by implementing the use of equivalent tidal volumes (ETV).^12^, ^24^ Using ETV assures that the volume change between the phases registered to compute the ventilation are similar for both the pre‐RT and post‐RT registration. This essential step mitigates changes in ventilation observed longitudinally due to changes in the breathing effort of the subject and allows us to attribute regional Jacobian changes to dosimetric or physiological factors. A result of using equivalent volumes is that regions with low pre‐RT function can show an increase in ventilation to compensate for regions of the lung with reduced ventilation due to radiation, in order for the whole lung to reach the same tidal volume as the pre‐RT scan. This volume normalization is necessary to assess regional changes, but it may mask global lung changes or potential lung damage. Another reason for low‐function regions to show an increase in post‐RT function is that there may be tumor regression reducing airway occlusion.

We found that high‐function lung regions sustain larger decreases in function as dose increases, when averaged over all 12 subjects. However, individual subjects may not follow this trend exclusively, as there are many confounding variables when measuring lung ventilation longitudinally. Du et al.^24^ used equivalent volumes to obtain the highest scan repeatability, with an average gamma pass rate of 76.3% between two pre‐RT scans for 13 subjects. This shows that even when there should be no ventilation change with the scans being 5–15 min apart, there are still some changes in the measurement of regional ventilation. This can be observed in the large error bars in Fig. 4. While we have reduced variation in patient respiratory effort between scans using audio cues to aid in respiratory normality and selecting equivalent volumes for registration, additional studies to improve the repeatability in ventilation measurements are needed to examine an individual's response to radiation.

The pre‐RT dependence of sensitivity to dose may be important for treatment planning if ventilation‐based functional avoidance is being used. The stratification of pre‐RT function into 6 discrete levels shows increased radiation‐induced functional decline with increased pre‐RT function. A voxel that we would consider high functioning with a pre‐RT Jacobian of 1.15, has an estimated decline in function of 1.0% per 10 Gy. However, another high‐function voxel with a pre‐RT Jacobian of 1.55 has an estimated decline in function of 4.8% per 10 Gy, which is 4.8 times higher than the other voxel. Therefore, a single high‐function region of interest to avoid during the planning process may be inadequate. Our study suggests multiple regions representing levels of pre‐RT function are indicated, as moderate variations in pre‐RT function can result in large sensitivity differences to radiation dose.

## 5 Conclusions

While SPECT is the most prevalent modality for measuring local lung function clinically, ventilation estimates derived from 4DCT datasets have been validated by multiple groups. Utilizing 4DCT datasets has the advantage of routine accessibility without an additional imaging procedure, and it provides a means to normalize patient effort variability during longitudinal studies using equivalent tidal volumes.

There are ongoing clinical trials that are incorporating 4DCT‐based functional avoidance into lung cancer treatments. Our results, showing that high‐function lung had a greater radiation‐induced reduction in ventilation compared to low‐function lung, support the hypothesis of these trials: avoiding high‐function lung regions during RT yields improved pulmonary function preservation.

## Conflicts of interest

The authors have no relevant conflicts of interest to disclose.
